# Milking of the Umbilical Cord in Term and Late Preterm Infants

**DOI:** 10.1155/2019/9185059

**Published:** 2019-02-11

**Authors:** Stefano Basile, Sara Pinelli, Elisabetta Micelli, Marta Caretto, Pierluigi Benedetti Panici

**Affiliations:** ^1^Maternal and Child Health Department, Division of Obstetrics and Gynecology 2, Pisa University Hospital, Pisa, Italy; ^2^Department of Gynecologic-Obstetrical and Urologic Sciences, Sapienza University Hospital, Rome, Italy

## Abstract

**Introduction:**

Umbilical cord milking is a procedure in which clamped or unclamped umbilical cord is grasped, and blood is pushed (“stripped”) two to four times towards the newborn, in a rapid time frame, usually within 20 seconds. The target of umbilical cord milking is to provide infants with their whole potential blood volume—of which they are deprived when early cord clamping is carried out—completing placental transfusion in a shorter time than delayed cord clamping. The aim of this narrative review is to analyse the literature regarding umbilical cord milking in term and late-preterm infants and to assess all possible benefits and limits of this procedure in clinical practice, especially in comparison to immediate and delayed cord clamping.

**Methods:**

We analysed literature data concerning maternal, as well as neonatal, outcomes for term and late-preterm (gestational age ≥ 34 weeks) newborns who received umbilical cord milking.

**Results:**

Most studies show comparable benefits for both umbilical cord milking and delayed cord clamping, especially in terms of haematological parameters when compared to immediate cord clamping. Umbilical cord milking may be a feasible procedure also for newborns requiring resuscitation.

**Conclusions:**

Literature data concerning positive effects of umbilical cord milking are encouraging and suggest that umbilical cord milking may be a quick and effective method to provide placental transfusions to depressed infants. However, the lack of standardised procedures and the variation in evaluated outcomes as well as the limited number of patients enrolled in trials, along with the retrospective nature of some of them, prevent recommending umbilical cord milking as a routine procedure.

## 1. Introduction

In the past, active management of the third stage of labour involved three subsequent processes: administration of a prophylactic utero-tonic therapy, Immediate Cord Clamping (ICC) and cutting, and controlled traction of the umbilical cord. This triad was envisaged by the World Health Organization (WHO) in the attempt of reducing the risk of Postpartum Haemorrhage (PPH), a major complication in the third stage of labour and the main cause of maternal morbidity and mortality worldwide [1–3].

While active management has proven to reduce the risk of PPH, evidence shows that, among the three procedures mentioned, only the prophylactic utero-tonic therapy actually reduces the risk of PPH [1–3]. Indeed, it is questionable whether ICC or cord traction affects the amount of blood loss at childbirth. Evidence also suggests that delayed cord clamping (DCC) (usually defined as clamping of the cord at least 30–60 seconds after birth) [4] may improve neonatal outcomes compared to ICC [5, 6]. According to these data, DCC would increase the level of placental transfusion to newborns, as it allows a longer time for transferring an additional 30% of blood volume, with up to 60% more red blood cells from placenta to infant at the time of birth [7–12].

The increased placental transfusion obtained with DCC leads to higher neonatal haemoglobin levels [4, 7–10], additional iron stores, higher red blood cell flows to vital organs, and lower anaemia occurrences later in childhood [13–15].

Given the benefits to most newborns linked to this procedure, the majority of professional and scientific organizations now recommend DCC for all vigorous term and preterm infants “if they are not depressed at birth” [16, 17] at least 30–60 seconds after birth, in absence of contraindications [4].

There may will be infants at risk of resuscitation, who could not wait 30-60 seconds and in such cases umbilical cord milking (UCM) was suggested to increase the speed of placental transfusion.

Cord milking may offer a substantial advantage over DCC in hypoxic newborns, who cannot wait for DCC as they would be at high risk of severe intraventricular haemorrhage and death [18].

UCM is the procedure defined as of 'stripping' the blood from the umbilical cord to the newborn in a rapid time frame, usually within 20 seconds [19, 20]. The term is used interchangeably for intact umbilical cord milking (I-UCM) (the cord is milked when it is still connected to the placenta) and cut-umbilical cord milking (C-UCM) (the cord is milked when it is cut and separated from the placenta).

I-UCM represents an alternative to DCC when the unclamped umbilical cord is grasped, and blood is pushed (“stripped”), before being clamped, from two to four times. Careful attention should be paid to how cord milking is performed, i.e., how many times and whether the cord is kept intact. Literature evidence shows that cord milking with an intact cord immediately improves pulmonary blood flow and assists lung expansion at breathing onset [4].

On the other hand, C-UCM, widely common in Asia, involves clamping and cutting 25 cm of cord segment from the umbilical stump immediately after birth [16]. The paediatric provider then takes the long cord, untwists it, and milks the entire contents into the baby.

During cord blood transfer to the infant, babies are usually held in a neutral position in relation to the placenta. To date, there are no studies identifying a recommended suitable position during UCM [21].

Placental transfusion obtained through DCC or UCM includes some theoretical risks, such as overtransfusion, polycythaemia, hyperbilirubinaemia, jaundice, or delayed resuscitation [22]. As a consequence, some resistance to these practices persists among midwives and medical staff, even if a recent meta-analysis suggests these concerns to be unfounded [20, 23].

Perrone and Ghirardello [24] put forward the first nationwide survey on placental transfusion strategies among tertiary-care delivery wards in all of the Italian territory. The results they obtained showed a low application rate for either DCC or UCM, especially at lower gestational ages, when placental transfusion is associated with improved systemic perfusion.

The aim of this narrative review is to analyse the literature concerning maternal as well as neonatal outcomes of UCM in term and late-preterm (gestational age ≥ 34 weeks) infants and to assess all possible benefits and limits of this procedure in clinical practice, especially in comparison to ICC and DCC.

## 2. Material and Methods

### 2.1. Search Strategy

We performed a review search up to 5th of January 2019 of PubMed database to identify eligible studies evaluating the role of UCM in term and late-preterm pregnancies (≥34 weeks).

We carried out a search using the following query: “umbilical cord milking”, and we screened 58 articles. A second search using the following query: “placental transfusion” turned out 176 articles.

We considered all studies analysing umbilical cord milking outcomes for term and late-preterm (gestational age ≥ 34 completed weeks gestation) newborns during the third stage of labour and relative maternal outcomes.

We considered both Caesarean Section (CS) and vaginal deliveries, due to the insufficient number of isolated CS in connection with cord milking in published data. We did not consider multiple pregnancies cases, due to the lack of sufficient specific data concerning such cases.

Among the founded articles, we analysed randomised clinical trials, retrospective, observational, and comparative studies on humans, whereas we excluded single case reports, reviews, and meta-analysis. We also excluded articles that were not written in English; articles covering inadequate patient populations (i.e., early preterm, animals); articles not evaluating UCM outcomes.

Then we analysed references from all included studies, in order to identify possible additional eligible studies, thus adding 1 article to our pool of relevant articles.

The final number of studies included in our review was 12.

## 3. Results (Table 1)

### 3.1. Maternal Outcomes

No maternal outcome was found in studies taking into account UCM procedures.

### 3.2. Neonatal Outcomes

#### 3.2.1. Birth Weight

No difference was reported regarding infant birth weight, neither among the UCM and ICC groups [16, 25] nor among the UCM and DCC groups [22, 26, 27].

#### 3.2.2. Infants Requiring Resuscitation/Respiratory Support

Regarding infants requiring resuscitation, in a quasirandomised, nonblinded, controlled trial, Girish et al. showed that UCM is feasible for term and late-preterm infants (≥35 weeks) who are depressed at birth. Compared to ICC, they found no significant difference in infants submitted to UCM in resuscitation delay, resuscitation efforts, and short-term outcomes [25]. No significant difference was found between infants of the UCM group and of the ICC group regarding Apgar scores at 1, 3, and 10 minutes, while the number of infants requiring chest compression, epinephrine, or fluid bolus was also similar among the two groups. For what concerned death rate, duration of hospitalisation, or inotrope use, no difference was observed among the two groups. Girish et al. then concluded that the use of UCM in depressed newborns does not delay resuscitation or adversely affects resuscitation efforts.

Katheria et al. conducted a retrospective analysis of data on about 157 term and late preterm infants (35–42 weeks) with abnormal cord blood gases after birth [22]. Compared to the ICC group, fewer infants who received UCM needed resuscitation (38% versus 56%, p = 0.07) and ongoing respiratory support (19% versus 31%, p = 0.16), although these rates did not reach statistical significance.

#### 3.2.3. Hypoxic-Ischemic Encephalopathy (HIE)

In the study by Girish et al. no significant difference was found in the number of infants with HIE in the UCM group (25 out of 50, 50%) and in the control group (26 out of 51, 51%) [25].

On the other hand, in Katheria's retrospective analysis fewer infants who received UCM showed evidence of HIE on magnetic resonance imaging postrewarming (5 days of life) when compared to the ICC group (8% versus 10%, p = 0.99), even if this rate was not statistically significant [22].

#### 3.2.4. Clinical Jaundice/Jaundice Requiring Phototherapy

Another significant aspect regarding UCM and DCC is the putative increase in the incidence of clinical jaundice subsequent to augmented placental transfusion, which is not confirmed by the published studies.

In this regard, Jaiswal and colleagues conducted a randomised controlled trial comparing the effect of UCM and DCC on haematological parameters in newborns (>36 weeks) at 6 weeks of life [27]. No significant difference was reported in this study in relation to the development of jaundice and the requirement of phototherapy among the two groups.

On the other hand, Erickson-Owens et al. enrolled 24 women undergoing elective term CS and their foetuses and randomised them to ICC or UCM. Also the results obtained by Erickson-Owens about the development of clinical jaundice or requirement for phototherapy were not statistically significant [23].

In another study, Upadhyay and colleagues investigated the effect of C-UCM as compared to ICC among term and near term (>35 weeks' gestation) in a randomised controlled study. The researchers observed no significant difference in serum bilirubin levels among infants in both groups, while none of the enrolled babies required phototherapy [16].

Yadav and colleagues in their randomised controlled trial compared the effects of DCC plus clamping the cut cord (DCM) with the effects of DCC or UCM alone in 300 term newborns [28]. Regarding jaundice, serum bilirubin level at 48 h and the number of patients requiring phototherapy for jaundice were comparable in all three groups.

#### 3.2.5. Haemoglobin Concentrations and Haematocrit Ratio

Regarding haematological parameters such as haemoglobin (Hb) concentration or haematocrit ratio, the evaluated studies seem to show a comparable beneficial effect for UCM and DCC, which appear both preferable to ICC.

In fact, in Jaiswal's study Hb and haematocrit after 30 minutes from birth resulted equal among UCM and DCC groups [27].

However, in the randomised controlled trial by Yadav, the mean Hb and haematocrit in first 30 min and 48 h were significantly higher in DCM group as compared with DCC and UCM group alone (P = 0.0001)[28].

Other studies evaluated Hb and haematocrit also at 36-48 hours of life. Erickson-Owens et al. found significantly higher Hb levels and haematocrit ratio at 36–48 hours in the UCM group than in the ICC group [23].

Similarly, concerning haematological parameters in the first days of life, Upadhyay showed that the mean Hb levels and haematocrit ratio at 12 hours and at 48 hours were significantly higher in the C-UCM group as compared to ICC group (P=.0001) [16].

Also a recent study by Alzaree and colleagues randomised two groups of term (≥ 37 weeks) neonates to receive UCM or DCC [29]. Those researchers found comparable levels of neonatal Hb on the first day among the two groups.

On the other hand, analysing haematological parameters at 6 weeks of life, no difference among UCM and DCC groups was observed in the trial by Jaiswal et al. in relation to Hb levels for term and near-term neonates (Hb 11 versus 11.3 gr/dl, 95% CI -0.46 to 0.94). The same result was obtained in the trial by Yadav et al., where the mean Hb at 6 weeks in all three groups (DCM, UCM, and DCC) were comparable [28].

On the contrary, comparing C-UCM and ICC, Upadhyay et al. found that mean Hb was significantly higher in the intervention group (C-UCM), than in the ICC group of the trial (11.9 gr/dl versus 10.8 gr/dl, respectively) on infants of 6 weeks of age [16]. Similarly, regarding the fetal Hb at 6 weeks, Alzaree and colleagues found a significantly higher Hb level in UCM group rather than in DCC group [30].

Bora and colleagues performed a basic intent-to-treat analysis, comparing 6-month serum ferritin and Hb levels of infants randomised to long umbilical cord and milking (the umbilical cord was clamped at 40 cm length and milked) and to ICC (the cord was clamped within 30 seconds of life at 5 cm from the umbilicus, but no milking was performed). Intervention group infants showed statistically significant higher mean Hb values compared to controls (9.61 gr/dl in the intervention group (95% CI 9.32 to 9.90) versus 9.07 gr/dl in the control group (95% CI 8.84 to 9.30), p= 0.004) [31].

Agarwal et al. designed a follow-up study comparing the effects of DCC at 60-90 seconds and UCM on haematological parameters in infants of 12 months of age. The study demonstrated that mean Hb levels were not significantly different in the 2 groups [32].

#### 3.2.6. Ferritin Concentrations

Iron stores at birth are crucial to a newborn's growth and on these depends the development of iron deficiency anaemia during childhood. Placental transfusion at the time of delivery, through DCC or UCM, can influence iron stores. Ferritin is the main iron storage protein in the human body. Despite being an intracellular protein, the concentration of ferritin in soluble form in the blood can determine an iron deficiency. Regarding ferritin levels in newborns, outcomes of different studies showed that results from UCM and DCC were comparable, while they were both more favourable compared to ICC.

In fact, while the trial by Upadhyay et al. [16] reported a significantly favourable effect on term and near-term neonates' serum ferritin from C-UCM compared to ICC at 6 weeks of life, Jaiswal [27] found comparable ferritin levels in neonates at 6-weeks of life among UCM and DCC groups.

Bora and colleagues specify that the mean value of serum ferritin was 70.8 ng/ml (95% CI 62.5 to 79.2) in the ICC group at 6 months of age, whereas it reached 113.9 ng/ml (95% CI 105.7 to 123.8) in the C-UCM group, with a high statistically significant difference (95% CI 31.7 to 56.1, p value 0.001) [31]. However, the study by Agarwal et al. showed that the mean serum ferritin in the DCC group (16.44 *μ*g/L) was comparable to that of the UCM group (18.2 *μ*g/L) at one year of age [32].

Interestingly, the study by Yadav founded a significantly higher serum ferritin at 6 weeks in DCM group than DCC and UCM group [28].

#### 3.2.7. Placental Transfusion Blood Volume

The only study evaluating the amount of blood transferred through UCM was carried out by McAdams et al. [29]. It enrolled 60 women at ≥37 weeks' gestation, whose babies received I-UCM or C-UCM procedure. Researchers measured blood volumes with graduated collection cups. This pilot study is interesting because it analyses the actual amount of blood transferred through the different procedures (DCC, C-UCM, I-UCM) in order to define a standard protocol for application in clinical practice.

The result the team came to was that in term newborns, I-UCM (×3 or ×4 stripping) produces higher blood volumes (BV) than through C-UCM. In C-UCM procedures, the longer the length of the cord segment, the higher the total milked BV obtained.

#### 3.2.8. Cerebral Blood Flow

Another interesting issue is how placental transfusion strategies can influence cerebral blood flow in newborns.

For the first time, in their randomised controlled trial, Jaiswal et al. evaluated Middle Cerebral Artery (MCA) blood flow velocity and Doppler indices through cranial ultrasound, between 24 to 48 hours from birth, along with haemodynamic parameters (like blood pressure, heart rate, respiratory rate) at 30 minutes, 24 hours and 48 hours of life in term infants, randomised to C-UCM or DCC. They remarked that cerebral blood velocities and cranial Doppler indices were similar in both C-UCM and DCC group. Similarly, there was no significant difference among the two groups in relation to mean resistive index (RI), pulsatility index (PI), and MCA cerebral blood flow velocity [26].

## 4. Discussion

Since recent evidence started to underline the importance of placental transfusion in neonatal outcomes, clinical trials analysed the role of each individual triad component promoted in the past by the WHO for the active management of the third stage of labour. As a consequence, the practice of immediate cord clamping was excluded by the WHO guidelines in 2012, and cord traction was defined as optional [1–3].

Placental transfusion is the transfer of placental blood to the infant during the first few minutes after birth [33]. This procedure is associated with lower rates of mortality in preterm infants and with the prevention of iron deficiency anaemia in term neonates [10, 32].

Placental transfusion can be performed through the traditional DCC or UCM and may represent an important procedure for ensuring the newborn a smooth transition to extra-uterine life. DCC and UCM can in fact both enhance arterial oxygen content and haemodynamic stability and can be easily provided also in low-resource settings. Cord clamping time, uterine contractions, umbilical blood flow, breathing, and gravity all play a central role in determining placental transfusion BVs.

DCC provides a passive transfer of placental blood, at a slow rate, while UCM involves an active stripping of blood through the umbilical cord to the newborn, at a faster rate [30].

The majority of studies on preterm infants have clearly demonstrated a comparable increase in haemoglobin levels after DCC and UCM. Furthermore, UCM was also associated with higher language and cognitive scores in very preterm infants when compared to DCC [33], whereas data concerning term and late-preterm infants are still insufficient to draw definite conclusions on cognitive neurodevelopment.

According to* RCOG Scientific Impact Paper*, 2015, UCM represents a valid alternative to DCC in case of preterm births, but it needs to be further investigated in order to evaluate associated benefits and risks before it can be performed routinely [18].

We should remember anyway that large randomised trials on UCM in high-income countries are insufficient and that there may be differences among the effects seen in preterm and term infants.

Moreover, there is no standardisation on DCC optimal time, despite time being essential for passive placental transfusion. As it is common practice to pass a depressed newborn over to the paediatric staff as soon as possible, the safety of newborns requiring resuscitation is yet to be established.

Also, the time needed for DCC passive placental transfusion might protract maternal bleeding and delay hysterotomy closing in case of CSs, or episiotomy or perineal tear repairing in vaginal deliveries [20].

According to Italian recommendations in case of CS term newborns, if DCC cannot be performed, UCM may be considered as an alternative procedure with the purpose of increasing haemoglobin levels in postnatal period and iron reserves in the following weeks [24]. In this scenario, this practice rapidity of execution may offer a significant advantage in reducing maternal blood loss.

A strong limitation of this review is the lack of trials analysing maternal outcomes. We had no basis for demonstrating alleged benefits in terms of blood loss. For this reason, further larger trials investigating this issue are necessary.

Among the limitations of this review, we acknowledge the manifold evaluated outcomes, varying widely across the studies, the limited number of patients enrolled in some of the mentioned studies, and also the retrospective nature of some of these. Another limitation is the lack of UCM standardisation across the different studies.

We think that premature and term newborns requiring resuscitation may be the most in need of a placental transfusion, benefiting from more blood returning to their body immediately after birth.

While it is possible to provide resuscitation during DCC, there are a number of logistical challenges particularly in the sterile operative field and in premature infants. Anaemia due to iron deficiency represents a major health problem, especially in low-income country infants. UCM could be a safe, feasible, inexpensive, and less time-consuming alternative to DCC in order to prevent neonatal iron deficiency anaemia.

In fact, UCM is believed to be a simple procedure that can be safely performed in a matter of seconds by obstetrical staff, with no long learning curve. Furthermore, this method may be very useful in cases of neonatal asphyxia, given the crucial importance of time in such situations. As showed by Girish et al., UCM may be a feasible procedure also for neonates requiring resuscitation, as it proved to not cause any resuscitation delays in depressed newborns compared to ICC [26]. Katheria et al. reached similar conclusions in their retrospective analysis regarding neonates with acidosis, as infants who had received UCM and needed resuscitation and ongoing respiratory support were fewer in number than those who received ICC [22].

Although all cited studies evaluating haematological parameters such as haemoglobin, haematocrit, and ferritin showed significantly higher UCM results compared to ICC groups at different times from the delivery [16, 23, 31, 32], DCC and UCM obtained comparable results [29].

Despite the fairly good number of studies evaluating UCM outcomes, a standardised procedure is needed. McAdams's is the only study evaluating different outcomes between different UCM procedures, showing that I-UCM (×3 or ×4) promotes a larger transfusion of blood volume to newborns at birth than C-UCM [30].

Even if published data on UCM positive effects are encouraging, suggesting that UCM may be the most effective method to provide placental transfusions in infants requiring resuscitation, evidence on which category of newborns would benefit the most from UCM is still inadequate [22].

Enhanced implementation of the procedure could be associated with clinical UCM guidelines availability, knowledge of UCM benefits, and strict cooperation within the delivery team.

The topic is of the utmost importance as it concerns the vast majority of newborns in diverse delivery settings worldwide.

In conclusion, the insufficient knowledge of placental transfusion limits and benefits leads to a wide variation in the management of cord clamping. It would then be useful to standardise the UCM procedure in order to offer protocols applicable to clinical practice, and to spread knowledge among professionals through educational programs.

## Figures and Tables

**Table 1:** 

**Author**	**Type of the study**	**Number of infants**	**Interventions**	**Primary outcomes**	**Results**
***Erickson-Owens, 2012***	Randomized controlled trial	24 in term (>37 weeks of gestation) infants delivered by elective CS	**UCM**: 12 **ICC**: 12	Hct levels at 36 to 48 h of age exposed to ICC or UCM	higher Hct levels at 36 to 48 h of age in UCM group

***Upadhyay, 2013***	Randomized controlled trial	200 term and near-term infants (≥35 weeks of gestation)	**C-UCM**: 100 **ICC**: 100	Hb and serum ferritin at 6 weeks of postnatal age	Mean Hb (11.9 [1.5] g/dL and mean serum ferritin 355.9 [182.6] *μ*g/L) significantly higher in the C-UCM group Vs. ICC group (10.8 [0.9] g/dL and 177.5 [135.8] *μ*g/L), respectively, at 6 weeks of age

***Jaiswal, 2015*** ***PMID: 25800496***	Randomized controlled trial	200 term infants (>36 weeks of gestation)	**C-UCM**: 100 **DCC**: 100	Serum ferritin and Hb at 6 weeks of life	Mean serum ferritin (134.0 ng/ml [89.8]) and mean hemoglobin (11.0 gm/dl [2.4]) in C-UCM group was comparable to mean serum ferritin (142.7 ng/ml [87.1]) and hemoglobin (11.3 gm/dl [2.6]) in DCC group at 6 weeks of age

***Jaiswal, 2015*** ***PMID: 26008758***	Randomized controlled trial	200 term infants (>36 weeks of gestation)	**C-UCM**: 100 **DCC**: 100	RI, PI and cerebral blood flow velocities of MCA at 24 to 48h of life	Mean PI [1.18 (0.26)] and RI [0.65 (0.08)] in UCM group was comparable to mean PI [1.18 (0.25)] and RI [0.65 (0.08)] in DCC group. The peak systolic velocity and end diastolic velocity (cm/s) of blood flow in MCA for UCM group were 34.94 (11.82) and 11.71 (4.75) respectively, while in DCC group they were 37.24 (12.63) and 13.07 (4.78) (p 0.23 and 0.07) respectively

***Bora, 2015***	Randomized controlled trial	Infants of non-anemic mothers (≥37 weeks of gestation): 90 Infants of anemic mothers: 89	Infants of non-anemic mothers: ICC 45 C-UCM 45 Infants of anemic mothers: ICC 44 C-UCM 45	Hb and serum ferritin concentrations at 6 months of age	Mean Hb concentration at 6 months was 9.60±1.42 g dl(-1) in the C-UCM group and 9.07±1.10 g dl(-1) in the ICC (P=0.004). Mean serum-ferritin concentration at 6 months was 113.9±43.8 ng ml(-1) in the C-UCM group and 70.8±39.5 ng/ml in the ICC (P<0.001)

***Yadav, 2015***	Randomized controlled trial	300 neonates (≥37 weeks of gestation)	UCM: 100 DCC: 100 DCM: 100	Hb and serum ferritin at 6 weeks of age	Median serum ferritin at 6 weeks was significantly higher in the DCM group (295.49 (233.5 to 346.54) ng ml− 1) Vs. UCM group (184.55 (131.22 to 256.5) ng ml− 1) or only DCC (268.8 (189.4 to 315.44) ng ml− 1).

***Agarwal, 2016***	Follow-up study of a randomized controlled trial	161 (≥37 weeks of gestation)	**UCM**: 83 **DCC**: 78	Hb and serum ferritin concentrations at 12 months of age	The mean Hb in the DCC group (102.2 (17.2) g/L and serum ferritin 16.44 (2.77) *μ*g/L) showed no significant differences to the UCM group (98.6 (17.1) g/L and 18.2 (2.8) *μ*g/L) at one year

***Katheria, 2018***	Retrospective analysis	157 infants with abnormal cord gases (≥35 weeks of gestation)	UCM: 36 ICC: 121	neonatal outcomes during hospitalization	Fewer infants in the UCM group needed resuscitation (38 vs. 56%, p = 0.07) and ongoing respiratory support (19 vs. 31%, p = 0.16) compared to the ICC

***McAdams, 2018***	Clinical comparative study	60 women ≥37 weeks' gestation	**I-UCM:** 15 3x Milkings 15 4x Milkings **C-UCM:** 10 Cut10cm, 10 Cut20cm 10 Cut30cm	Placental transfusion blood volumes	Mean blood volume with I-UCM (×4) was increased compared to the 30 cm C-UCM technique (48.5 ± 19.0 vs. 24.8 ± 4.0 mL, P < 0.001). For C-UCM, blood volume increased proportionally to cord length and, by the second milking, 98.1 ± 4.5% of blood volume was delivered.

***Alzaree, 2018***	Randomized controlled trial	250 (≥ 37 weeks' gestation)	**DCC: **125 **UCM: **125	Infant's Hb at 6 weeks after delivery	There was a significant statistical difference between cases delivered by UCM and those delivered by a DCC in Hb after 6 weeks

***Girish, 2018***	Quasi-randomized, non-blinded, controlled trial	101 infants (≥35 weeks) who were depressed at birth	**NO UCM**:51 **UCM**: 50	Feasibility and safety of UCM	No significant differences in resuscitation delay, resuscitation efforts, and short-term outcomes between the two groups.

Hct= haematocrit.

Hb= haemoglobin.

CS= cesarean section.

RI= resistive index.

PI= pulsatility index.

MCA= middle cerebral artery.

UCM= umbilical cord milking.

DCC= delayed cord clamping.

DCM= DCC with milking the cut cord.
